# Fused Particle Fabrication 3-D Printing: Recycled Materials’ Optimization and Mechanical Properties

**DOI:** 10.3390/ma11081413

**Published:** 2018-08-12

**Authors:** Aubrey L. Woern, Dennis J. Byard, Robert B. Oakley, Matthew J. Fiedler, Samantha L. Snabes, Joshua M. Pearce

**Affiliations:** 1 Department of Mechanical Engineering–Engineering Mechanics, Michigan Technological University, Houghton, MI 49931, USA; alwoern@mtu.edu (A.L.W.); djbyard@mtu.edu (D.J.B.); 2 re:3D Inc., 1100 Hercules STE 220, Houston, TX 77058, USA; robert.oakley@re3d.org (R.B.O.); matthew@re3d.org (M.J.F.); samantha@re3d.org (S.L.S.); 3 Department of Material Science and Engineering, Michigan Technological University, Houghton, MI 49931, USA; 4 Department of Electrical and Computer Engineering, Michigan Technological University, Houghton, MI 49931, USA; 5 Department of Electronics and Nanoengineering, School of Electrical Engineering, Aalto University, 00076 Espoo, Finland

**Keywords:** 3-D printing, additive manufacturing, distributed manufacturing, open-source, polymers, recycling, waste plastic, extruder, upcycle, circular economy

## Abstract

Fused particle fabrication (FPF) (or fused granular fabrication (FGF)) has potential for increasing recycled polymers in 3-D printing. Here, the open source Gigabot X is used to develop a new method to optimize FPF/FGF for recycled materials. Virgin polylactic acid (PLA) pellets and prints were analyzed and were then compared to four recycled polymers including the two most popular printing materials (PLA and acrylonitrile butadiene styrene (ABS)) as well as the two most common waste plastics (polyethylene terephthalate (PET) and polypropylene (PP)). The size characteristics of the various materials were quantified using digital image processing. Then, power and nozzle velocity matrices were used to optimize the print speed, and a print test was used to maximize the output for a two-temperature stage extruder for a given polymer feedstock. ASTM type 4 tensile tests were used to determine the mechanical properties of each plastic when they were printed with a particle drive extruder system and were compared with filament printing. The results showed that the Gigabot X can print materials 6.5× to 13× faster than conventional printers depending on the material, with no significant reduction in the mechanical properties. It was concluded that the Gigabot X and similar FPF/FGF printers can utilize a wide range of recycled polymer materials with minimal post processing.

## 1. Introduction

With the introduction of the self-replicating rapid prototyper (RepRap) 3-D printer [1,2,3], the costs of additive manufacturing (AM) with 3-D printers have been reduced enough to be accessible to consumers. This has allowed for the emergence of a distributed manufacturing paradigm in AM [4,5,6], where 3-D printing can be used to manufacture products for the consumer and by the consumer directly, with significant savings compared to the purchasing of mass-manufactured products [7,8,9,10,11,12]. The emerging support for this model in the business literature [13,14,15] is in part due to the exponential rise of free digital design file sharing for 3-D printed products [12], which ranges from sophisticated scientific instruments [16,17,18,19,20] to everyday toys for children and cosplayers [10]. Regardless of the sophistication of the product, high return on investments (ROIs) can be enjoyed based on the download substitution values using commercial polymer 3-D printing filament [21,22]. Commercial filament, however, is marked up significantly (e.g., >5×–10×) over the cost of the raw polymers, which limits the cost savings and thus, the deployment velocity of distributed manufacturing to further increase the rate of accessibility of AM [23]. The negative effects of the high costs of filament are most notable for large format 3-D printers (those with a build volume that is greater than a cubic foot), which can process several kg of polymer in a single print lasting over 24 h.

One method of overcoming these cost barriers is to use a means of distributed plastic recycling, involving upcycling plastic waste into 3-D printing filament with a recyclebot (an open source waste plastic extruder [24]). Previous research on the life cycle analysis of the recyclebot process found a 90% decrease in the embodied energy of the filament from the acquiring, processing of the natural resources, and the synthesizing compared to traditional filament manufacturing [25,26,27]. This allows for the tightening of the loop of the circular economy [28] because it enables real distributed recycling that eliminates nearly all energy use and pollution from transportation. Many recyclebot versions have been developed including open source variations from the Plastic Bank, Filastruder, Precious Plastic, Lyman, and Perpetual Plastic, as well as fully commercial versions including the Filastruder, Filafab, Noztek, Filabot, EWE, Extrusionbot, Filamaker (also has a shredder), and the Strooder, Felfil (OS) [29]. Most recently, a “RepRapable Recyclebot” has been demonstrated [30], where most of the machine’s parts can be 3-D printed from waste plastic themselves. Several polymers have been successfully recycled as single component thermoplastic filaments, such as polylactic acid (PLA) [30,31,32,33,34], high-density polyethylene (HDPE) [24,35,36], acrylonitrile butadiene styrene (ABS) [28,36,37], elastomers [9], as well as composites (e.g. waste wood [38] and carbon fiber reinforced [39]).

Unfortunately, each time a polymer is heated and extruded (whether it be in the recyclebot filament making process or during conventional fused filament fabrication (FFF)/fused deposition modeling (FDM) 3-D printing), the mechanical properties are degraded [31,32,40,41]. This effectively limits this process of recycling to 5 cycles [31,32] without the use of blending virgin materials or adding materials for mechanical property reinforcement. To reduce the number of melt/extrude cycles of recycled plastic that is used for 3-D printing, one option is to eliminate the need for filament and print directly from pellets, flakes, regrind, or shreds of recycled plastic, which will be referred to as particles here. Several 3-D printers using fused particle fabrication (FPF) (or fused granular fabrication (FGF)) have been designed to accomplish this in the academic [42,43,44], hobbyist [43,44,45], and commercial systems [46,47,48,49,50]. These systems have been tested with virgin pellets, however the mechanical properties of FPF printers using recycled polymer particles of various shapes and sizes has not been reported, which limits the ability of engineers to fabricate load bearing products from recycled waste using FPF 3-D printing.

In this study, the open source Gigabot X [51], which is a large scale recycled plastic 3-D printer, was used to fabricate and test the mechanical properties of parts that were made using FPF to fill this knowledge gap. To establish a baseline, virgin PLA pellets were analyzed first and were then compared to four recycled polymers: (1) PLA regrind of 3-D printed parts (the most common 3-D printed plastic), (2) recycled ABS pellets (the second most common 3-D printed plastic), (3) recycled polyethylene terephthalate pellets (PET, the most common waste plastic [52]), and (4) recycled polypropylene chips (PP, the second most common waste plastic [52]). First, the material size characteristics were quantified using digital image processing. Then, a power and nozzle velocity matrix printing test were completed to determine the optimum print speed and temperature settings for a given polymer feedstock. During this phase of testing, any problems with bed adhesion and warping were identified and resolved. Third, a set of ASTM type 4 tensile bars were printed and were pulled to confirm the mechanical properties of the plastic when it was printed with a pellet drive extruder system and was compared with the results of past work with FFF/FDM 3-D printers, while noting the number of melt cycles.

## 2. Materials and Methods

### 2.1. Materials

Virgin 4043D PLA pellets were obtained from Nature Works LLC. PLA regrind was obtained from a mixture of failed 3-D prints from various sources of filaments and was ground with an open source grinder [53]. PP regrind was provided by McDonnough Plastics. Northwest Polymers and CiorC provided ABS and PET recycled materials. The size characteristics of the particles for each starting material were quantified using digital imaging and the open source Fiji/ImageJ [54].

### 2.2. FPF 3-D Printer

**Figure 1:**
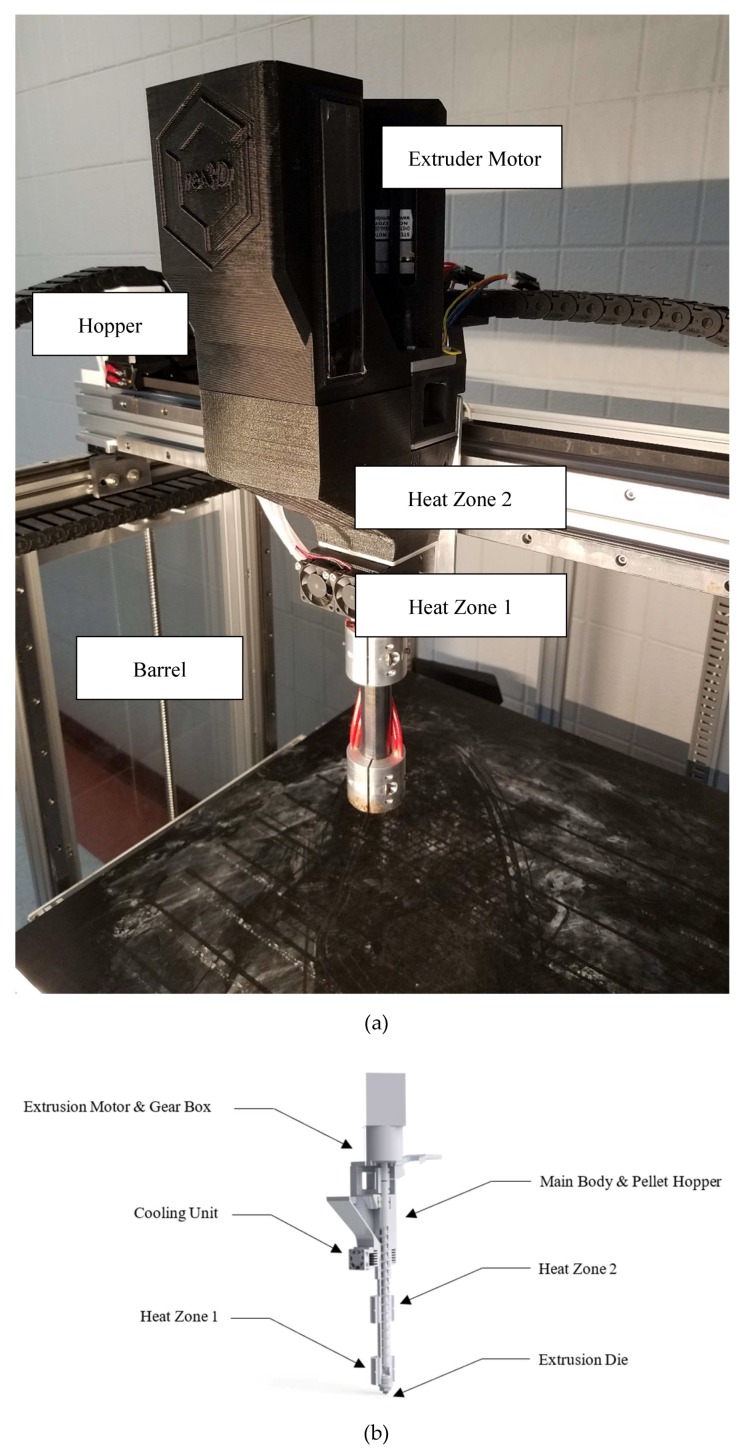
(**a**) The open source Gigabot X with the major components labeled. (**b**) Details of the extruder.

A prototype Gigabot X [55] was used to print the materials. The prototype used an extruder system that was closely related to an industrial thermoplastic extruder, however it was scaled down and mounted as the extruder head of the 3-D printer. The extrusion screw was designed with an increasing diameter down the length of the screw with a ratio of 2.5:1 from the start to the end [55]. The motor driving the extrusion screw was a NEMA 23 stepper motor with a planetary gearbox with an 18:1 reduction. This ran off an external stepper driver [55]. The hopper was 3-D printed to allow for ease of modification and optimization of the design during testing. The extruder was heated by four 60-watt 24-volt heater cartridges that were placed in groups of two in strategic locations on the barrel [55]. Figure 1a shows a Gigabot X (the parts are labeled along with the heating zones) and Figure 1b shows the details of the extruder in a cutaway computer aided design (CAD) model with the major components labeled. Full details of the design can be found at reference [55]. 3-D models were sliced with Slic3r [56] and the printer was controlled with Marlin Firmware [57]. The testing procedures were then developed for an FPF platform to allow for the data to be collected efficiently for each plastic. These data can be collected in a couple of working days and provide information on the optimum settings regardless of what type of plastic is used. This is beneficial for expediting the continued work on this or similar platforms with new polymers, additives, and composites.

### 2.3. Optimization of Temperature and Speed 

The power vs. velocity matrix testing was accomplished by first identifying the upper and lower bounds of the printable temperature range of a given plastic through literature research, as summarized in Table 1.

**Figure 2:**
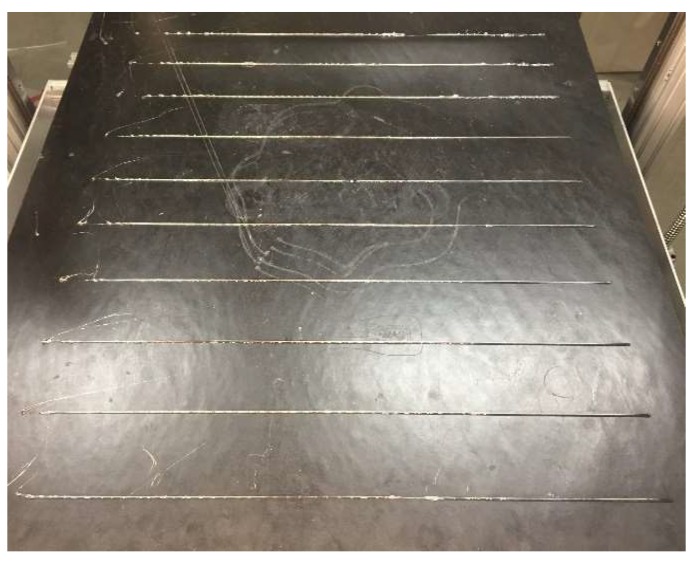
Materials Sample Matrix Test (polylactic acid (PLA) shown).

Heating zones 1 (tip of the nozzle) and 2 inside the barrel were then set to every combination of temperatures in the printable temperature range at an appropriate resolution to capture the entire spectrum. A series of double lines were then printed at each temperature combination at print speeds from 5 to 50 mm/s in 5 mm/s increments [62]. The printed lines (example in Figure 2) were then massed on a digital scale (+/− 0.01 g) and the line masses were compared to the theoretical line mass. The objective of this test was to find both the optimum print speed and the temperature settings for the extruder for each 3-D printing material. The optimum settings were determined both by the temperature settings that had the lowest standard deviation in terms of the line weight for a set of lines and the print speeds across all of the temperature settings, which resulted in the heaviest line masses.

A single walled vase test [63] was used to determine the ideal settings for the flow percentage and the actual extrusion width once the print speed and the temperature zone settings had been confirmed. To conduct this test, a single walled hollow cylinder with known theoretical dimensions was printed. Once the print was completed, the specimen’s mass was recorded and was compared to the theoretical mass of the component using the specific density of the plastic that was used. The method of similar triangles was then used to determine the appropriate flow percentage. Once the flow percentage was successfully calibrated, digital calipers (+/−0.005) were used to find the printed wall thickness. This value was then loaded into the slicing software (Slic3r) so that the printed line width could be corrected for in the slicing software.

### 2.4. Tensile Testing

Tensile testing was first completed on PLA using the ASTM D638 Type 1 and Type 4 standard tensile bars. For PLA, Types 1 and 4 were used to examine any potential difference in mechanical performance due to the smaller geometry of the Type 4 tensile test bars, as has been done before with more conventional FFF based 3-D printing [64]. This was a point of concern due to the relatively large size of the printer nozzle diameter (1.75 mm) compared to the smallest dimension of the Type 4 tensile bars (2 mm). The remainder of the tensile testing was completed using ASTM D638 Type 4 standard tensile bars. The bars were printed at ideal print settings that were found during matrix testing at 100% infill. The infill pattern was set to 0 degrees or “perpendicular” with respect to the long axis of the tensile bars. The samples were then pulled until failure using a 5000 lb. load cell (Model LCF455). The strain data was captured using a 1-inch Epsilon extensometer (Model E95691, Error: +/−0.0276 mm).

## 3. Results

### 3.1. Materials Size Distribution

**Figure 3:**
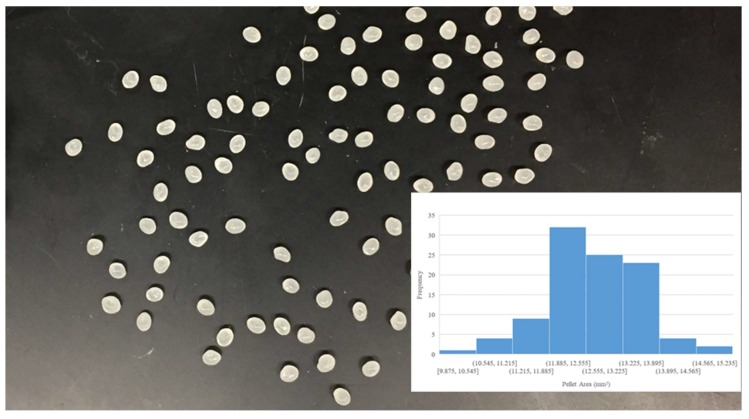
Virgin PLA pellet size distribution.

**Figure 4:**
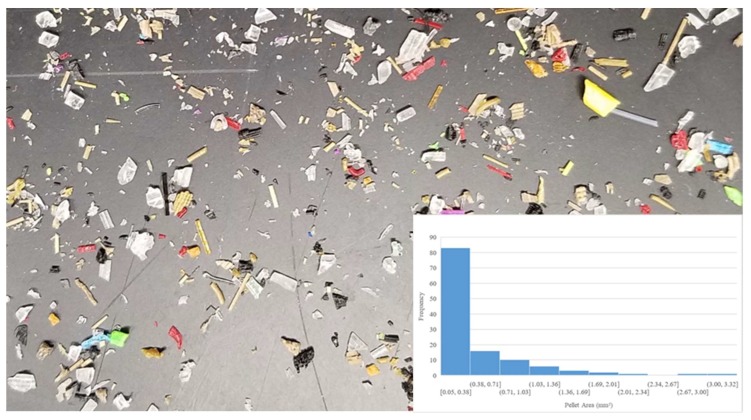
Reground 3-D printed PLA size distribution.

**Figure 5:**
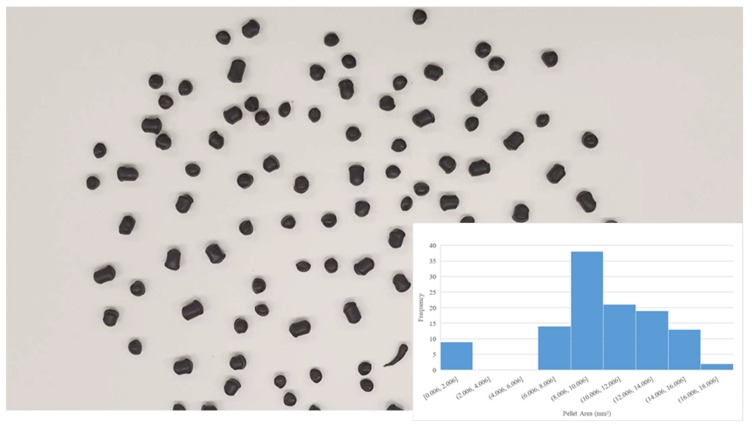
Recycled acrylonitrile butadiene styrene (ABS)) pellet size distribution.

**Figure 6:**
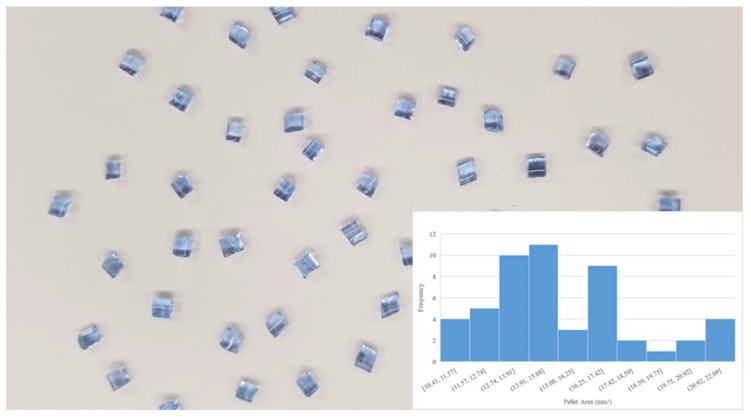
Recycled as well as the two most common waste plastics (polyethylene terephthalate (PET) size distribution.

**Figure 7:**
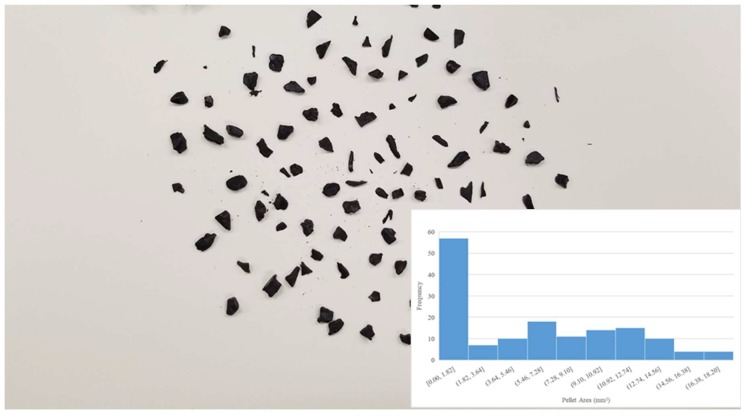
Recycled polypropylene (PP) flake size distribution.

Digital images of the input polymer materials are shown in Figure 3, Figure 4, Figure 5, Figure 6 and Figure 7 for virgin PLA, recycled PLA, ABS, PET, and PP, respectively. In each Figure, the inset shows the particle size distribution for the material. The virgin PLA had the most uniform particles as well as the most spherical shape which provided the best feeding. The recycled polymers, however, had much larger size distributions. The regrind polypropylene and regrind PLA were the least consistent. Both of the reground plastics consisted of flakes, dust-like particles, and large chunks, all of which surprisingly fed nicely into the auger system without jamming or miss-feeding. It was concluded that pellets with areas smaller than 22 mm^2^ could feed into the auger regardless of shape.

### 3.2. Optimal Printing Temperatures and Velocities

For each polymer, a matrix was generated for the range of the operating temperatures for the two temperature zones as well as the nozzle velocities from 5 to 50 mm/s. The difference between the theoretical and the actual mass of the line is shown as a function of the nozzle speeds. The color coding on the charts represent how different each actual line mass is compared to its ideal mass. The farther away from the ideal, the redder the number becomes. For the main part of the chart, zero would be ideal, and the farther away from zero, the worse the sample was and the redder it is. For the average and standard deviation section, the more green the number is, the closer the average was to its ideal mass. The standard deviation tells us how much each mass varied with one another. The ideal temperature combination is one that has a low average difference and a low standard deviation.

#### 3.2.1. Virgin PLA

**Figure 8:**
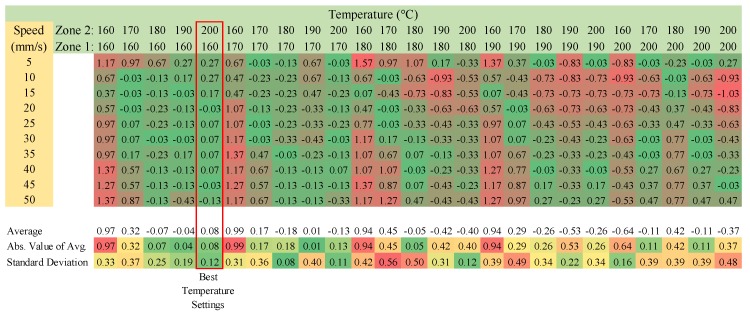
PLA Virgin-Difference between the theoretical and actual mass of the line-speed temperature matrix.

Figure 8 shows the results for virgin PLA. The ideal temperature settings were found to be 160 and 200 °C for heat zones 1 and 2, respectively. In examining the general trend across the matrix, also note that higher zone 1 temperatures, with respect to zone 2, results in a higher standard deviation for a given line test. This has been corroborated by initial literature research [65]. This phenomenon is attributed to the specific density differential of the plastic across zones 1 and 2, creating a stabilizing back pressure at the extrusion die.

**Figure 9:**
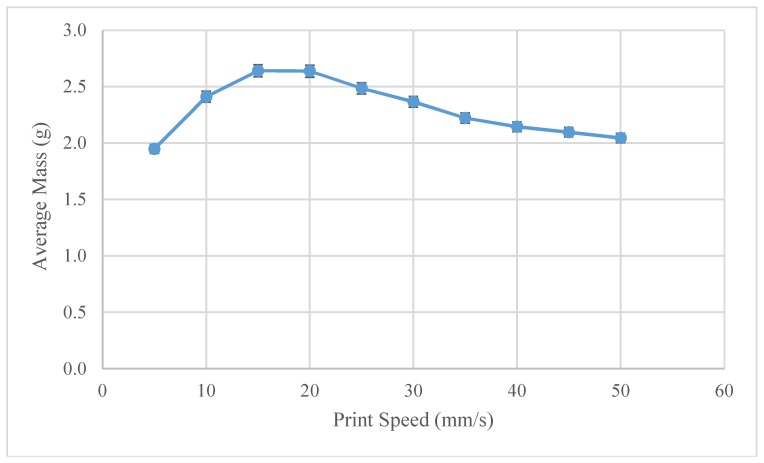
Virgin PLA–Average mass as a function of the print speed.

Observing the trend in the average line weight speeds across the full temperature spectrum, line weight is maximized at print speeds of around 20 mm/s, as shown in Figure 9. At this print speed, the volumetric deposition rate of the printer is 35 mm^3^/s. In comparison, a traditional FFF printer at a print speed of 50 mm/s, with print settings of 0.2 mm layer height with a 0.4 mm nozzle, will average at 4 mm^3^/s. This translates to an 8.75x increase in the speed of the 3-D printed part formation.

#### 3.2.2. Reground 3-D Printed Parts PLA

The reground PLA was printed with the optimized settings that were found for virgin PLA. It printed well considering it had different types, colors, sizes, and shapes of particles being fed into it. The extruder did a good job of mixing the polymer inside the auger because the prints came out a consistent dark green color. Mechanical testing was conducted and it was found that the recycled PLA had a similar tensile strength to the virgin PLA, with the average being 38 MPA compared to 39 MPA for the virgin. This compares to studies by Cruz Sanchez et al. [31,32] where they showed that the tensile strength of PLA degraded by about 2% after the first cycle. It was found that the tensile strength difference between the recycled PLA and the Virgin PLA that was printed on the Gigabot X was about 2.5%.

It was discovered that there was little difference printing reground PLA versus printing with virgin PLA. Using the same optimum settings resulted in tensile strengths that would be expected from the additional melt/print cycle [31,32].

#### 3.2.3. Recycled ABS Pellets

**Figure 10:**
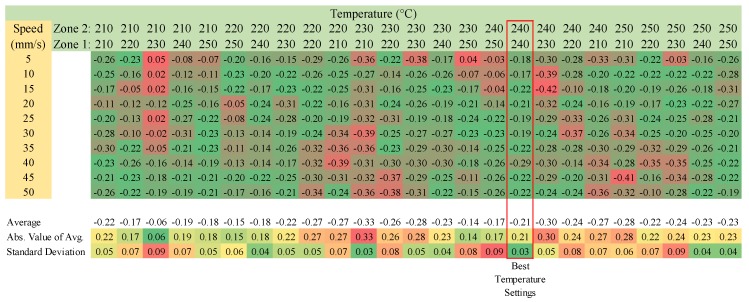
The Recycled ABS-Difference between the Theoretical and the Actual Mass of the Line-Speed Temperature Matrix.

Approximately 3 mm recycled ABS pellets were verified for printing with FPF type 3-D printers. The results of the speed matrix testing are shown in Figure 10. Temperature settings of 240 °C and 240 °C were identified as the optimum temperature settings for heating zones 1 and 2 on the printer, and the bed temperature was set to 90 °C.

**Figure 11:**
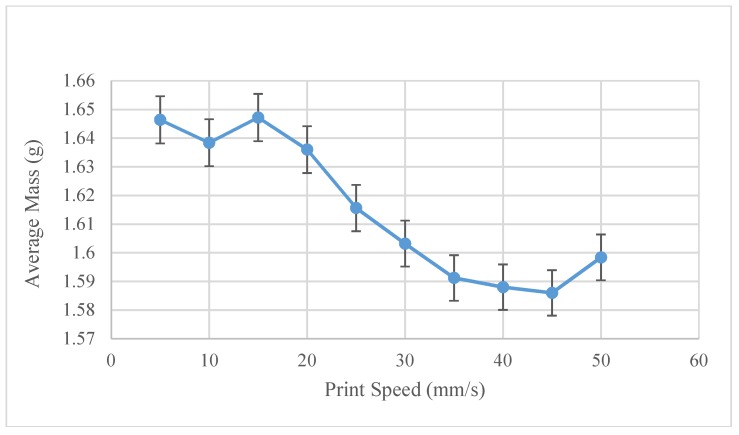
Recycled ABS-Average mass as a function of print speed.

The optimum print speed settings were experimentally found to be 15 mm/s, as can be seen in Figure 11. It should be noted, however, that the total range of the line weights shows minimal insensitivity to the print speed, as is noted by the smaller range (1.57–1.64 g) of line weights. At 15 mm/s, the volumetric flow is 26.25 mm^3^/s. Comparing this speed to a normal FFF printer at 4 mm^3^/s, the FPF system can print 6.5× faster in ABS.

#### 3.2.4. Recycled PET Pellets

**Figure 12:**
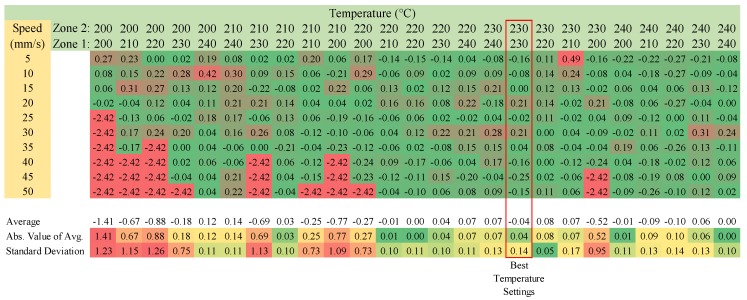
Recycled PET-difference between the theoretical and the aActual mass of the line-speed temperature matrix.

Material testing was conducted on ~4 mm recycled PET pellets. The results of this testing can be found in Figure 12. It should be noted that the ideal temperature settings of 220 °C and 230 °C were identified for zones 1 and 2. A bed temperature of 100 °C was found to achieve the best adhesion results.

**Figure 13:**
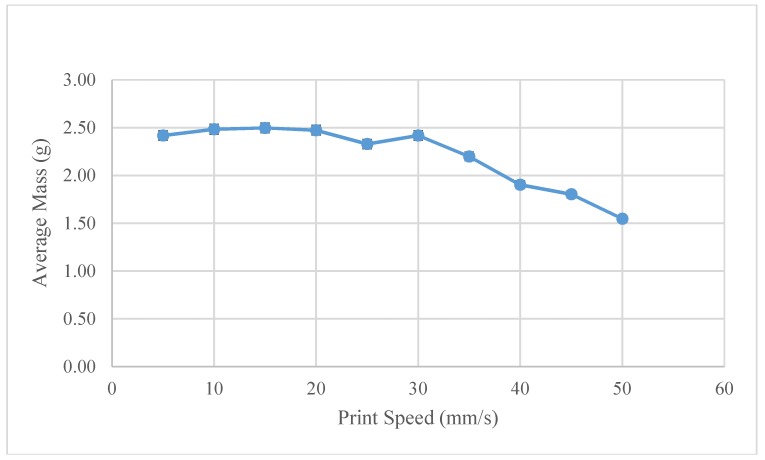
PET-Print speed effect on the printed line.

Figure 13 shows that when printing with recycled PET, speeds between 5 mm/s and 30 mm/s have little difference in terms of average mass. When printing past 30 mm/s however, the mass substantially drops and, in some cases, when the temperatures were low, the extruder motor would skip and stall, causing the print to fail.

#### 3.2.5. Recycled PP Chips

**Figure 14:**
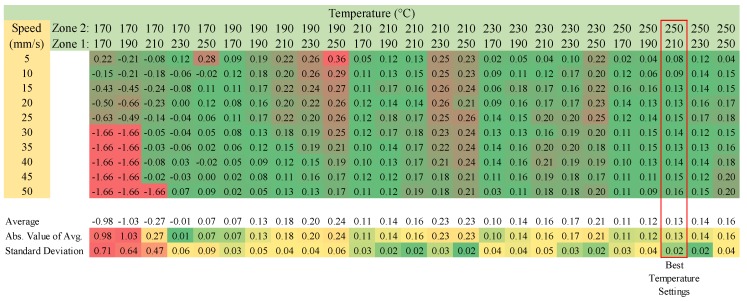
Reground PP-Difference between the theoretical and the actual mass of the line-speed temperature matrix.

Reground PP chips were used to verify PP as a valid print material. The results of the matrix testing can be found in Figure 14, where the optimum print temperatures were found to be at 230 °C and 250 °C for heat zones 1 and 2, respectively.

When examining Figure 14, it should be pointed out that failures occurred when printing at too low of a temperature. The extruder motor stalled because of the high viscosity of the plastic at those temperatures. To prevent damage to the machine, the test was halted at the first sign of an extruder stall.

**Figure 15:**
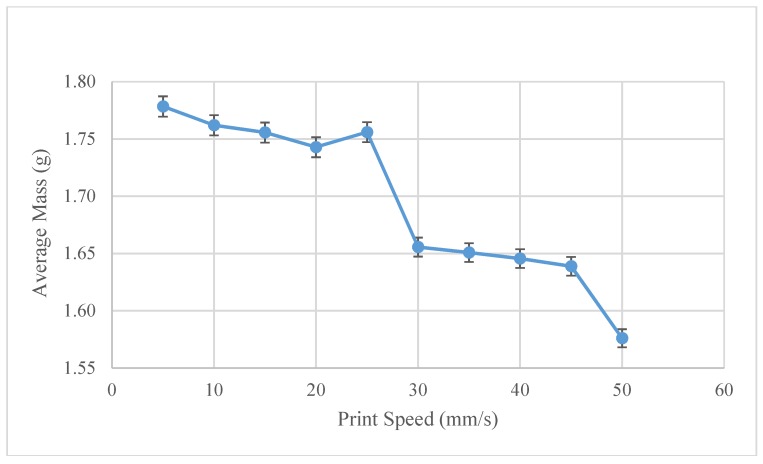
Recycled PP-Average mass as a function of print speed.

As seen in Figure 15, average line masses as a function of print speed, 30 mm/s was identified as the optimum print speed. Although, it should be pointed out that considering the overall magnitude of the mass scale, the weight is relatively insensitive to the print speed. At these settings, however, the volumetric printing speed is about 52.5 mm^3^/s, or has a deposition rate that is ~13 times faster than traditional FFF printing.

### 3.3. Mechanical Testing

The mechanical properties for PLA that is printed with FPF are comparable to what has been reported in standard FFF type printers where the tensile strength was found to be 47.55–50.23 MPa [66]. The average strain at the break (mm/mm) was 0.02 with a standard deviation of 0.01 for Type 1 and 0.02 with a standard deviation of 0.01 for Type 4. In addition, the modulus (MPa) was 2815 for Type 1 (SD 166.4) and 2135 (SD 273.5) for Type 4. Notably, peak stress for the Type 2 and Type 1 tensile bars was 43.35 (+/−5.89) and 39.43 (+/−5.22) MPa, which is close enough to consider the Type 4 tensile bars as being representative of the printed mechanical properties here. Recycled ABS had a peak stress of 26.40 MPa (SD 1.78), recycled PET had a peak stress of 40.63 MPa (SD 2.41), and recycled PP had a peak stress of 23.82 MPa (SD of 0.49).

PLA has a bulk tensile strength of 16 to 103 MPa [67], whereas 3-D printed PLA is 56.6 MPa [68], 50–57 MPa, depending on the color [69]. In this study, it was found that the virgin PLA Tensile Strength was on average 39 MPa, which is comparable to the average that was found with FFF/FDM style printers. The lower than average result can be explained by the large nozzle size of the Gigabot X and the fact that it cannot print small objects that are less than 20 mm by 20 mm without introducing gaps in complex geometries. The tensile bars had visible holes at the neck region where the printer would skip over because the geometry was too tight for the slicer to print it. This resulted in breaks happening outside of the extensometer, near the neck region of the bar, corrupting any elastic modulus data.

ABS has a bulk tensile strength of 20 to 73 MPa [70], whereas 3-D printed ABS ranged from around 25 to 32 MPa [71], 28.5 MPa [68]. In this study, the recycled ABS was found to have an average tensile strength of 26 MPa. This falls into the range of FFF/FDM style printers, meaning that printing from granules does not directly reduce the tensile strength.

PET has a bulk Tensile Strength of 47 to 90 MPa [72], whereas 3-D printed PET ranged from 27 to 45 MPa [73]. In this case, recycled PET was printed and the testing concluded that the average tensile strength was 40 MPa. Comparing this to FFF printed PET, it is actually on the high end, meaning that the recycled nature of the plastic did not have any significant impact on the strength of the material.

PP has a bulk tensile strength of 4 to 369 MPa [74], whereas 3-D printed PP ranged from 27 to 36 MPa [75]. PP was shown to have an average tensile strength of 24 MPa when it was printed with the Gigabot X. This is on the low end of both the bulk tensile strength and the FFF 3-D printed tensile strength, which could be explained by having more thermal cycles from numerous recycling processes and the purity of the PP, which are unknown with this specific plastic. This underscores the need for better material ingredient tracking [76] and expanded recycling symbols [77] as the recycling of 3-D printed parts becomes more widespread.

## 4. Discussion

The advantages of printing with recycled particles rather than filament include lower costs, because of the lower cost of the starting material, and it is also easier to print large objects (e.g., where more than 1 spool of material is required) [78]. This has caused a recent surge in research that is related to 3-D printing with pellets including new printer designs: using a double stage screw [79], derivative of a metal injection molder [80,81], those based on RepRap technology [82], and industrial robots for 3-D printing [83], as well as a large range of polymers including conventional filament materials and recycled materials [82] as well as conductive polymer composites [84] and flexible materials [85]. These studies all indicate that FPF 3-D printing will play a larger role in the future of the 3-D printing industry and are corroborated by the results of this study.

The Gigabot X successfully printed with a wide range of particle sizes and distributions, which opens up a large array of starting materials beyond high-quality (e.g., uniform size and spherical) pellets. It can be concluded that the Gigabot X and similar FPF printers with good auger-barrel tolerances (+/−0.025 mm) can handle a wide range of polymer inputs, including recycled materials with minimal post processing (i.e., only cleaning and grinding/shredding). In addition, due to the ease of extrusion and the ability to print in a wide distribution of particle sizes, this system is an ideal candidate for upcycling waste plastics and the development of unique plastics co-polymers and composites. The mechanical testing using tensile strength indicated that FPF did not degrade the polymer properties (e.g., similar to FFF), however, future work should also consider testing other mechanical properties such as compression, impact resistance, fracture toughness, creep testing, fatigue testing, and flexural strength. This will result in additional potential applications of recycled polymer FPF.

The ability to define multiple heating zones in the FPF extrusion system as in the Gigabot X is useful for multiple reasons. First, by establishing a pre-heating zone prior to extrusion, this helps to both transfer the required thermal energy to the plastic (given the high material flow rate) and to achieve sufficiently low viscosities for printing. The low viscosity also reduces motor wear from over torqueing and allows for faster material throughput during purging cycles. Another benefit of having multiple heating zones is to allow for more custom temperature profiles. Some plastics experience more shear heating than others. Therefore, having a descending temperature gradient would allow the temperature at the inlet to be higher to start melting and mixing the pellets as soon as possible to allow for the best throughput, while simultaneously setting the die heater at a lower temperature to prevent degradation from overheating in the shearing (metering) section. Changing the screw geometry or the compression ratio for every plastic would be very unsustainable, so being able to have control over how the different plastics flow while they are inside the extruder without having to replace the screw, comes down to controlling speed and temperature profiles.

The large format FPF type printer has unique abilities to print large components at high mass flow rates, resulting in dramatically shorter print times for large components. This is due to the ability to easily print with large nozzle sizes (1–2 mm). This is particularly useful in the manufacturing of large, functional components with non-critical surface smoothness criterion. Some applications include custom furniture, complete sporting goods equipment, large research tools, breathable casts and other health care products, agricultural processing equipment, OEM components, construction applications, and on demand fixtures or jigs.

There are, however, some limitations with this prototype system, including lower than normal FFF resolution in the x-y-plane (1.75 mm diameter minimum due to the nozzle diameter) and further limitations are imposed on the component size due to the high heat transfer rates from the large (comparative to traditional FFF printers) contact area of the printer’s hot-end, meaning that parts that are less than 20 mm by 20 mm cannot be printed reliably. Useful future work would consider other mechanisms for providing FPF beyond the system that has been described here. The Gigabot X is also currently lacking in any sort of part cooling system, which is a common feature on most FFF printers to assist in the cooling of the extruded plastics. This allows the printer to print complex geometries, such as overhangs of plastic, without losing dimensional accuracy or creating visual blemishes on the surface of the print. Without this, Gigabot X is limited in the geometries that it is capable of printing accurately. Finally, the prototype provides approximately six hours of continuous feed before manual replenishment. If this technology is to be scaled commercially, users would require much longer printing sessions and an automated feeding system.

Future work is needed to quantify the environmental benefits of using FPF over conventional FFF/FDM with both conventional filament as well as recycled filament. Several plastic pellet types and sizes from multiple vendors were used in this research. Significant variability in the size and the uniformity of the sourced pellets was observed between the different vendors. Further research on this hardware is needed to support supplier variability using non-uniform flake and/or pellets. Finally, a detailed cost analysis is needed to quantify the economic benefits of utilizing this approach.

## 5. Conclusions

The Gigabot X system presents a uniquely robust open source solution to large format FPF printing. Notable with this design was the ability to print a large variety of polymers at dramatically lower print times when compared to traditional FFF type printing. As recycled plastic and pellets are less costly than filament, this system succeeds in lowering the economic barriers to the fabrication of large format, high value, plastic components, which has been an unfulfilled gap in the open source, distributed manufacturing design space.

The tensile strengths of the printed parts for traditional FFF/FDM prints and the FPF prints are comparable for all of the polymers that were tested when the melt/print cycles are taken into account. This means that there will be no need to sacrifice part strength by using FPF systems. More studies need to be conducted to determine how the layer adhesion compares between an FFF and an FPF system.

Finally, a novel methodology was tested to successfully operate any FPF system (and the Gigabot X in particular) to experimentally determine the best 3-D printing parameters for new polymers. The line test was developed to show the best temperatures to print the new polymer regardless of type or particle size distribution. The single walled vase test was used to determine the theoretical vs actual mass and to get a flow percentage that would calibrate the extruder. The vase test was also used to determine the actual extrusion width to be put into Slic3r to prevent overlapping lines or under-extrusion. These simple tests can be performed over the course of a couple days for any unknown polymer and, once completed, should give the correct settings for successful prints.

## Figures and Tables

**Table 1:** 3-D printable temperature range for the two most common 3-D printed polymers and the two most common post-consumer waste polymers.

Material	Temperature Range °C	Source
PLA	160–200	[58]
ABS	200–250	[59]
PET	200–240	[60]
PP	170–250	[61]
